# Increased IL-26 associates with markers of hyperinflammation and tissue damage in patients with acute COVID-19

**DOI:** 10.3389/fimmu.2022.1016991

**Published:** 2022-11-17

**Authors:** Eduardo I. Cardenas, Sandra Ekstedt, Krzysztof Piersiala, Marianne Petro, Agneta Karlsson, Åsa Kågedal, Susanna Kumlien Georén, Lars-Olaf Cardell, Anders Lindén

**Affiliations:** ^1^ Division of Lung and Airway Research, Institute of Environmental Medicine, Karolinska Institutet, Stockholm, Sweden; ^2^ Division of ENT Diseases, Department of Clinical Science, Intervention and Technology, Karolinska Institutet, Stockholm, Sweden; ^3^ Department of ENT Diseases, Karolinska University Hospital, Stockholm, Sweden; ^4^ Karolinska Severe COPD Center, Department of Respiratory Medicine and Allergy, Karolinska University Hospital, Stockholm, Sweden

**Keywords:** coronavirus disease 2019 (COVID-19), severe acute respiratory syndrome coronavirus 2 (SARS-CoV-2), IL-26, IL-8, TNF, cd47, neutrophils, IL-6

## Abstract

Interleukin-26 (IL-26) is released by several immune and structural cells following stimulation of toll-like receptors (TLRs), whereupon it can directly inhibit viral replication and enhance neutrophil chemotaxis. Given these unique properties, IL-26 has emerged as an intriguing mediator of host defense in the lungs. However, the role of IL-26 in COVID-19 has not been thoroughly investigated. Here, we characterized the involvement of IL-26 in the hyperinflammation and tissue damage that occurs in patients with acute COVID-19. We found that IL-26 is markedly increased in blood samples from these patients, and that the concentration of IL-26 correlates with those of the neutrophil-mobilizing cytokines IL-8 and TNFα, respectively. Moreover, the increase in blood IL-26 correlates with enhanced surface expression of the “don’t eat me” signal CD47 on blood neutrophils isolated from patients with acute COVID-19. Finally, we found that the blood concentration of IL-26 correlates with that of increased lactate dehydrogenase, an established marker of tissue damage, and decreased mean corpuscular hemoglobin (MCH), a previously verified hematological aberration in COVID-19, both of which are associated with severe disease. Thus, our findings indicate that increased systemic IL-26 associates with markers of hyperinflammation and tissue damage in patients with acute COVID-19, thereby forwarding the kinocidin IL-26 as a potential target for diagnosis, monitoring, and therapy in this deadly disease.

## Introduction

Messenger RNA vaccines encoding for the SARS-CoV-2 spike protein have proven effective at reducing the number of severe cases of COVID-19 (1). However, the emergence of new variants of concern (2), limited vaccine distribution (3), and waning immunity (4) remain major problems that highlight the need for more effective therapies against the acute manifestations of COVID-19. Such therapies, in turn, require a deeper understanding of the cellular and molecular mechanisms underlying this disease. Moreover, it is now known that patients with acute COVID-19 present increased blood levels of pro-inflammatory cytokines (e.g., IL-6, IL-8, TNFα) that drive neutrophil mobilization and are associated with poor prognosis in severe cases (5, 6). Although neutrophils may contribute to viral clearance *via* cytokine release, phagocytosis, and production of extracellular traps (NETs), a growing body of evidence indicates that excessive neutrophil mobilization becomes pathogenic in COVID-19 (7). Along these lines, we and others recently demonstrated that blood neutrophils from patients with COVID-19 are hyperactivated and display enhanced survival and migration (6, 8). In addition, this excessive neutrophil mobilization has been confirmed by the increased markers of NET production found in blood from patients with acute COVID-19 (8, 9).

Until now, IL-26 has emerged as an intriguing mediator of host defense in lung disorders due to its antibacterial and neutrophil-mobilizing properties (10–16). However, it may also possess anti-viral potential; IL-26 was recently shown to bind viral RNA intermediates and inhibit the replication of the Hepatitis C virus, a virus that, just like SARS-CoV-2, contains a positive-sense single-stranded RNA genome (17). In addition, we have previously shown that IL-26 acts *via* its receptor complex (IL-10R2/IL-20R1) to enhance the IL-8- and fMLP-mediated chemotaxis of neutrophils *in vitro* (10), as well as the LPS-dependent accumulation of neutrophils in the lungs of mice *in vivo* (13). Furthermore, we and others have forwarded evidence that IL-26 alone or bound to NETs triggers the production of the neutrophil-related cytokines IL-6, IL-8, and TNFα in unsorted bronchoalveolar lavage (BAL) cells (10) and peripheral blood mononuclear cells (PBMCs) (18). Finally, we have shown that activation of TLR2, TLR3, TLR4, TLR7, and TLR8, all of which mediate SARS-CoV-2 recognition (19–23), elicits the release of IL-26 in human primary bronchial epithelial cells (TLR3, TLR7, and TLR8) (11), primary lung fibroblasts (TLR4) (24), alveolar macrophages (TLR4) (10), blood neutrophils (TLR4) (12), and the alveolar epithelial cell line A549 (TLR2 and TLR4) (12).

Despite its inherent antiviral and neutrophil-mobilizing properties, the involvement of IL-26 in COVID-19 has not been investigated in a conclusive manner. Therefore, in the current pilot study, we quantified the concentration of IL-26 in plasma samples from patients with acute COVID-19 (COVID-19 group), compared with healthy control subjects (Control group), and analyzed its association with the neutrophil-mobilizing cytokines IL-6, IL-8, and TNFα. We then characterized how the concentration of IL-26 associates with different markers of neutrophil activation, migration, survival, and NET production. Finally, we also determined the correlation between the plasma concentration of IL-26 and different markers of tissue damage and inflammation, as well as hematological parameters relevant to COVID-19. In doing so, we obtained strong evidence that the kinocidin IL-26 is increased at the systemic level and associates with several markers of hyperinflammation and tissue damage in acute COVID-19.

## Methods

### Patient material

All PCR-positive patients admitted between June 2020 and January 2021 to the COVID-19 subunit of the ENT department at the Karolinska University Hospital (Huddinge) were eligible for inclusion. At the time, the wildtype and alpha SARS-CoV-2 variants dominated in Sweden (25, 26). A healthy control group was recruited for comparison during the same period. Blood was collected at a single time-point and all participants provided written informed consent before sample collection. All procedures and handling of patient information were conducted in accordance with the ethical permit approved by the Swedish Ethical Review Authority in Gothenburg (Diary No. 2020-02579). A subgroup of this patient material was described in a recent publication (8).

### Quantification of cytokines and markers of NET formation

The presence of NETs in plasma was determined by measuring the concentration of double-stranded DNA (dsDNA) in a Quibit™ 3.0 Fluorometer (Thermo Fisher), and the levels of histone-complexed DNA (i.e., cell-free nucleosomes) using the Cell Death Detection ELISA^PLUS^ (Roche). The protein concentrations of IL-6, IL-8, IL-26, and TNFα in human plasma were quantified using ELISA (IL-6, IL-8, and TNFα: R&D Systems; IL-26: Cusabio) according to the manufacturer’s instructions. Cytokine concentrations were measured in samples from all patients and controls, but the plasma concentration of dsDNA and cell-free nucleosomes could only be measured in 32 out of 49 patients from the COVID-19 group, and 26 out of 27 participants from the Control group. Results on the specific levels of dsDNA, cell-free nucleosomes, IL-6, IL-8, and TNFα were already available from a previous publication on this patient material (8). However, all data on IL-26, as well as all comparisons presented in the current study have not been published elsewhere.

### Neutrophil isolation and flow cytometry

To isolate neutrophils from heparin-containing whole blood samples, red blood cells were eliminated *via* incubation with lysis buffer (0.8 mM NH_4_Cl, 10 mM KHCO_3_ 0.1 mM EDTA). The remaining cells were washed with PBS, stained with antibodies against CD11b, CD15, CD45, CD47, CD49d, and CD66b, and fixed (1% paraformaldehyde in PBS). Flow cytometry was performed on an LSRFortessa™ X-20 (BD-Biosciences) and neutrophils were defined as SSC^Int^ FSC^Int^ CD15^+^ cells. This analysis was performed in samples from 26 out of 49 patients in the COVID-19 group, and 12 out of 27 participants in the Control group. Results on the specific expression of all these markers for this patient material were already available from a previous publication (8). However, all comparisons presented in this study have not been published elsewhere.

### Quantification of hematological parameters and markers of inflammation and tissue damage

Blood concentrations of lactate dehydrogenase (LDH), C-reactive protein (CRP), and procalcitonin (PCT), as well as the mean corpuscular hemoglobin (MCH) and cell blood counts were determined using routine methods at the clinical laboratory of the Karolinska University Hospital. Information on MCH could only be retrieved from 31 out of 49 patients in the COVID-19 group.

### Statistical analysis

Non-parametric statistical analyses were performed in Prism 9.3 (GraphPad). Pairwise comparisons were assessed by unpaired, two-tailed Mann-Whitney test. Associations between two continuous variables were determined *via* Spearman rank’s correlation test. A p-value ≤ 0.05 was considered significant.

## Results

### Patient characteristics

Forty-nine hospitalized patients with PCR-confirmed SARS-CoV-2 infection and 27 healthy control subjects were enrolled in the study. The main characteristics of enrolled patients and controls are summarized in **Supplementary Table 1** (see also “Methods” for more information). The median age, as well as the number of male subjects, tended to be higher in the COVID-19 group. Most patients (44 out of 49) included in the COVID-19 group had severe disease (hospitalized with supplemental oxygen).

### Increased plasma concentration of IL-26 in acute COVID-19

**Figure 1:**
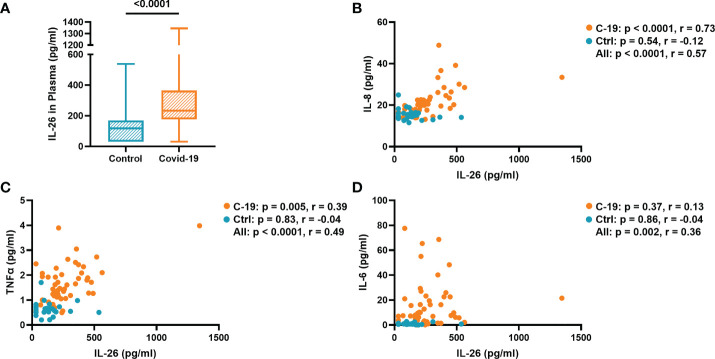
The plasma concentration of IL-26 is associated with those of IL-8, TNFα, and IL-6. **(A)** Comparison between the plasma concentration of IL-26 in the COVID-19 and Control groups tested by unpaired Mann-Whitney test. Spearman correlation analyses of the plasma concentration of IL-26 with those of **(B)** IL-8, **(C)** TNFα, and **(D)** IL-6 in the COVID-19 (orange) and Control (blue) groups.

The concentration of IL-26 in plasma was significantly increased in the COVID-19 group compared to the Control group (**Figure 1A**). Previously, we had shown that the plasma concentrations of IL-6, IL-8, and TNFα were similarly increased in this patient material (8). To better understand the relationship between IL-26 and these other cytokines, we now performed correlation analyses and found that the concentration of IL-26 displayed a strong positive correlation (r = 0.73) with that of IL-8 (**Figure 1B**), and a modest positive correlation (r = 0.39) with that of TNFα (**Figure 1C**), in the COVID-19 group. Moreover, the IL-26 concentration displayed a modest positive correlation (r = 0.36) with that of IL-6 when all the subjects in the COVID-19 and Control groups were pooled together (**Figure 1D**). Importantly, we did not detect any statistically significant differences regarding gender for any of these cytokines within the COVID-19 or Control groups (**Supplementary Figures 1A–D**). Furthermore, we did not detect a statistically significant correlation between the plasma concentration of IL-26 and age within the COVID-19 or Control groups (**Supplementary Figures 2A, B**). Notably, the concentration of IL-26 was still increased in a statistically significant manner in the COVID-19 group compared to the Control group when age matched subjects (30-61 years of age) were investigated (**Supplementary Figures 2C, D**).

### Higher plasma concentrations of IL-26 and IL-8 are associated with increased neutrophil survival in acute COVID-19

**Figure 2:**
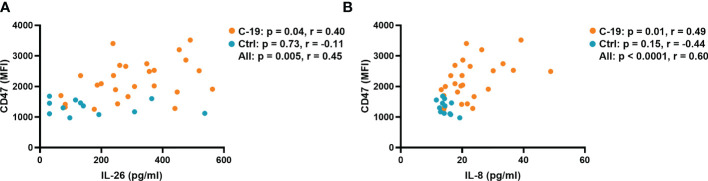
The plasma concentrations of IL-26 and IL-8 are associated with the surface expression of CD47 on blood neutrophils in acute COVID-19. Spearman correlation analyses of the plasma concentrations of **(A)** IL-26 and **(B)** IL-8 with the surface expression of CD47 on blood neutrophils from the COVID-19 (orange) and Control (blue) groups.

We have recently shown that blood neutrophils isolated from patients in the COVID-19 group have a higher surface expression of the “don’t eat me” signal CD47, which prevents phagocytosis and prolongs survival (8). Interestingly, among the cytokines investigated, only IL-26 (r = 0.40) and IL-8 (r = 0.49) displayed a positive correlation with CD47 expression in blood neutrophils from the COVID-19 group (**Figures 2A, B**). The plasma concentration of TNFα correlated with the surface expression of CD47 when the COVID-19 and Control groups were pooled for analysis, whereas the concentration of IL-6 in plasma did not correlate with CD47 expression in any group (**Supplementary Figures 3A, B**).

Moreover, we have recently shown that blood neutrophils isolated from the COVID-19 group have increased markers of activation (i.e., CD11b and CD66b) and a reduced surface expression of the maturity/migration marker CD49d (8). In the present study, we found that although the concentration of IL-26 did not correlate with the surface expression of CD11b in any group, it correlated with the surface expression of CD66b and the percentage of CD49d^+^ neutrophils when the COVID-19 and Control groups were pooled for analysis (**Supplementary Figures 4A–C**). Similarly, the concentrations of IL-6, IL-8 and TNFα correlated with the surface expression of CD11b and CD66b in the pooled analysis (**Supplementary Figures 5A–F**), whereas only IL-8 and TNFα (but not IL-6) correlated with the percentage of CD49d^+^ neutrophils when the COVID-19 and Control groups were combined for analysis (**Supplementary Figures 6A–C**).

### Plasma concentrations of IL-8 and TNFα are associated with NET markers in acute COVID-19

**Figure 3:**
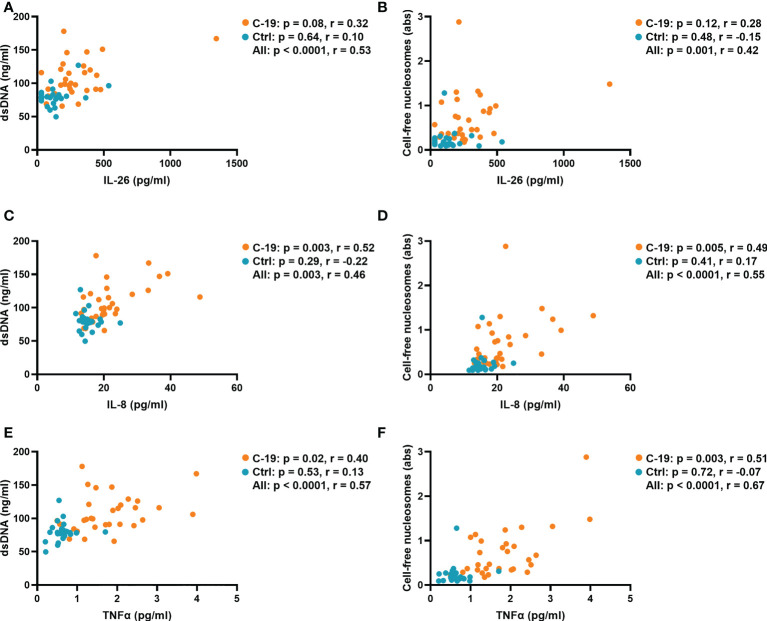
The plasma concentrations of IL-8 and TNFα are associated with those of NET markers in COVID-19 patients. Spearman correlation analyses of the plasma concentrations of IL-26, IL-8 and TNFα with the plasma concentration of **(A, C, E)** double-stranded DNA (dsDNA) and the plasma levels of **(B, D, F)** cell-free nucleosomes from the COVID-19 (orange) and Control (blue) groups.

Because IL-26 can bind and enhance the inflammatory potential of extracellular DNA (27), we characterized the relationship between the plasma concentration of IL-26 and markers of NET formation in the COVID-19 and Control groups. Notably, we and others have previously shown that NETs are increased in blood from COVID-19 patients (8, 9). Indeed, in the current study, we found that the plasma concentration of IL-26 correlates with those of double-stranded DNA (dsDNA) and cell-free nucleosomes when the COVID-19 and Control groups were pooled together for analysis (**Figures 3A, B**). In agreement with their known roles as inducers and/or enhancers of NET formation (28, 29), the plasma concentrations of IL-8 and TNFα correlated with those of both markers of NET production in the COVID-19 group (**Figures 3C–F**). On the other hand, the plasma concentration of IL-6 only correlated with that of cell-free nucleosomes, but not with dsDNA in the COVID-19 group (**Supplementary Figures 7A, B**).

### Plasma concentration of IL-26 correlates with that of lactate dehydrogenase in COVID-19 patients

**Figure 4:**
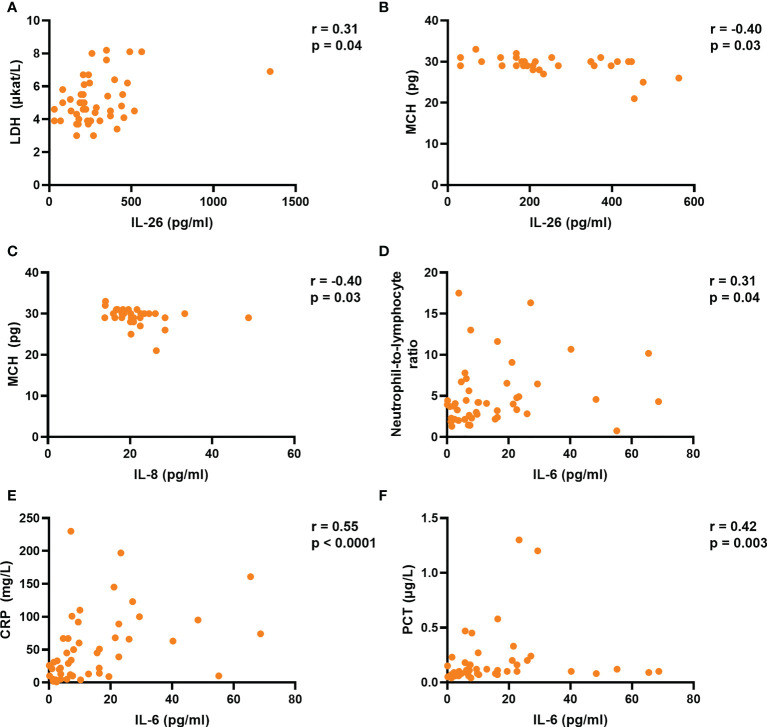
The plasma concentration of IL-26 is associated with those of lactate dehydrogenase (LDH) and mean corpuscular hemoglobin (MCH) in acute COVID-19. Spearman correlation analyses of the plasma concentrations of IL-6, IL-8, and IL-26 with those of **(A)** LDH, **(B, C)** MCH, **(D)** blood neutrophil-to-lymphocyte ratio, **(E)** C-reactive protein (CRP), and **(F)** procalcitonin (PCT) in the COVID-19 group.

Finally, we determined whether the plasma concentrations of IL-6, IL-8, IL-26, and TNFα correlate with markers of tissue damage and inflammation, as well as different hematological parameters and hospitalization time. Notably, we found that the concentration of IL-26 correlated in a positive manner with that of lactate dehydrogenase (LDH), a marker of tissue damage, in plasma from the COVID-19 group (**Figure 4A**). Moreover, the concentrations of IL-8 and TNFα (but not IL-6) tended to display a similar trend that failed to reach statistical significance (**Supplementary Figure 8A–C**). In addition, the plasma concentrations of both IL-26 and IL-8 displayed a negative correlation with the mean corpuscular hemoglobin (MCH) in whole blood from the COVID-19 group (**Figures 4B, C**), while IL-6 and TNFα failed to correlate with MCH in this way (**Supplementary Figures 9A, B**). On the other hand, only the plasma concentration of IL-6 correlated with the blood neutrophil-to-lymphocyte ratio and the plasma concentrations of C-reactive protein (CRP) and procalcitonin (PCT) (**Figures 4D–F**; and **Supplementary Figures 10A–C** and **11A–F**). Finally, only the plasma concentration of IL-8 displayed a statistically significant, albeit moderate (p = 0.32), positive correlation with hospitalization time in the COVID-19 group (**Supplementary Figures 12A–D**).

## Discussion

Although several previous studies have associated excessive neutrophil mobilization with poor prognosis in patients with acute COVID-19 (6, 8, 30), ours is the first to show that an increase in the plasma concentration of IL-26 correlates with signs of neutrophil mobilization in this disease. From an immunological point-of-view, it is feasible that IL-26 is involved in acute COVID-19, given its dual role as an antiviral and neutrophil-mobilizing mediator of host defense. It is known that the expression of IL-26 occurs constitutively in several immune and structural cells, and that it is enhanced upon TLR stimulation (31). Moreover, the genetic material of SARS-CoV-2 is recognized by TLR3 (21), TLR7 (21, 22), and TLR8 (22), critical receptors that are known to mediate the production of IL-26 in primary human bronchial epithelial cells (11). Furthermore, the SARS-CoV-2 spike protein can bind and activate TLR4 in a way that is comparable to LPS (19, 23). This may prove to be crucial, given that we have previously shown that exposure to LPS elicits a pronounced increase in IL-26 production in the lungs of healthy volunteers *in vivo* (10), as well as in blood neutrophils, primary human lung fibroblasts, and alveolar macrophages *in vitro* (10, 12, 24). Finally, the SARS-CoV-2 envelope protein is known to bind and activate TLR2 (20), which has been shown to cause the release of IL-26 in A549 cells, a cell line derived from human alveolar epithelial cells (12).

The relationship between IL-26 and the neutrophil-mobilizing cytokines IL-6, IL-8, and TNFα that we found in the current study is likely to be multifaceted. On the one hand, it is known that similar stimuli (e.g., TLR4 stimulation) can trigger the concomitant production of IL-6, IL-8, IL-26, and TNFα (31, 32). On the other hand, IL-26 alone or bound to NETs can induce the production of IL-8 and TNFα in unsorted BAL cells (10) and IL-6 in PBMCs (18), while inhibiting the production of the same cytokines in primary bronchial epithelial cells (11). Furthermore, it has previously been shown that TNFα together with IL-1β elicit the expression of IL-26 in human primary arterial smooth muscle cells (27), and that IL-26 enhances the IL-8-mediated chemotaxis of neutrophils isolated from human blood (10). Now, in the current study, we found that the enhanced plasma concentration of IL-26 displays a strong correlation with that of IL-8, and a somewhat weaker one with that of TNFα, in the COVID-19 group. Moreover, we found that IL-8 and IL-26 display comparable correlations with CD47, a marker of neutrophil prolonged survival, in the COVID-19 group. Thus, it seems possible that IL-26 is more functionally related to IL-8 than to IL-6 or TNFα.

Interestingly, although it has previously been shown that IL-26 enhances the pro-inflammatory potential of NETs and other forms of extracellular DNA (18, 27), we now failed to prove a correlation between the plasma concentration of IL-26 and the investigated markers of NET formation within the COVID-19 group. This was the case even though IL-8 and TNFα displayed such correlations. Nevertheless, we observed that the plasma concentration of IL-26 displays a trend towards a positive correlation with the concentrations of dsDNA (p = 0.08) and cell-free nucleosomes (p = 0.12) in the COVID-19 group, and it seems feasible that statistical significance could have been reached if the study material had included a larger number of patients in the COVID-19 group. However, we did prove a positive correlation between the plasma concentration of IL-26 and those of both markers of NET formation when the COVID-19 and Control groups were pooled, a finding that suggests a mechanistic link between IL-26 and NET production, possibly representing normal immunology rather than a unique pathological feature. If such an immunological mechanism does exist, it further implicates IL-26 in antimicrobial host defense in a wider sense, given the risk for bacterial infection that normally follows the damage of mucosal surfaces caused by viral infections.

Among the different markers of inflammation and tissue damage analyzed in the COVID-19 group, we proved a positive correlation between the plasma concentration of IL-26 and that of LDH, an established marker of tissue damage (33). Notably, increased serum LDH is known to be associated with severe COVID-19 (34, 35), and a recent study demonstrated that the serum concentration of LDH correlates in a positive manner with the degree of lung injury in patients with acute COVID-19 (34). In addition, we found that the plasma concentration of IL-26 is associated with decreased MCH, a hematological aberration detectable in mild, and further decreased in severe, cases of COVID-19 (36, 37). Taken together, these findings are suggestive of a mechanistic link between IL-26 and severe COVID-19 that deserves further investigation in larger study materials.

It is true that a previous study by Caterino M. et al. (38) failed to detect differences in the serum concentrations of IL-26 among COVID-19 patients with mild, moderate, or severe disease. However, their study did not include healthy control subjects. We think that their uncontrolled approach contributed to the failure to obtain evidence for the involvement of IL-26 in COVID-19. Furthermore, the fact that Caterino M. et al. studied only 27 patients in total, and as few as 6 with severe disease, argues that low statistical power made it impossible to detect differences among sub-groups with varying disease severity. Similarly, the limited size of our current study material, and the fact that it only included 5 patients with mild COVID-19 (hospitalized without supplemental oxygen), can explain why we failed to detect a statistically significant difference in the plasma concentrations of IL-26 between COVID-19 patients with mild or severe disease (p-value > 0.99, not shown), or a direct correlation between the plasma concentration of IL-26 and an assessment of disease severity (i.e., number of hospitalization days per patient). Moreover, we think that methodological aspects played a role in the study by Caterino M. et al., because they measured IL-26 in serum, whose generation requires blood coagulation—a likely confounder for cytokine release—and found that the concentration of IL-26 was below their technical limit of detection in 20 out of 27 patients. These results are in sharp contrast with our assessment of IL-26 in plasma, in which only 2 out of 49 patients in the COVID-19 group had a concentration of IL-26 below the technical detection limit.

In summary, our current pilot study forwards evidence that systemic IL-26 is markedly increased in patients with acute COVID-19, and that it correlates with neutrophil-mobilizing cytokines, a marker of prolonged neutrophil survival, and with markers of tissue damage and hematological alteration, the latter of which are known to signify severe COVID-19. Thus, IL-26 is involved in acute COVID-19, and it seems feasible that this intriguing kinocidin plays an important role in the hyperinflammation associated with acute COVID-19, a possibility that motivates further investigation into the clinical potential of IL-26 as a target for diagnosis, monitoring, and therapy in this deadly disease.

## Data availability statement

The original contributions presented in the study are included in the article/**Supplementary Material**. Further inquiries can be directed to the corresponding author.

## Ethics statement

The studies involving human participants were reviewed and approved by the Swedish Ethical Review Authority in Gothenburg (Diary No. 2020-02579). The patients/participants provided their written informed consent to participate in this study.

## Author contributions

EIC, SE, SKG, L-OC, and AL designed the outline of the study. AK, ÅK, and L-OC collected the patient material. SE, KP, and MP performed the neutrophil isolation, flow cytometry evaluation, and measurement of markers of NET formation. EIC, SE, KP, and MP measured blood cytokines. EIC and SE analyzed and compiled the collected data and performed all statistical analyses. SKG, L-OC and AL interpreted and supervised the data and statistical analyses. EIC and AL drafted the original manuscript that was read and reviewed by all authors. All authors contributed to the article and approved the submitted version.

## Funding

This work was financially supported by the Swedish Asthma Allergy Foundation (AL: # F2019-0031), Swedish Research Council (AL: # 2021-01527; L-OC: # 2016-01234) and Region Stockholm (AL: ALF # 2018-0088).

## Conflict of interest

The authors declare that the research was conducted in the absence of any commercial or financial relationships that could be construed as a potential conflict of interest.

## Publisher’s note

All claims expressed in this article are solely those of the authors and do not necessarily represent those of their affiliated organizations, or those of the publisher, the editors and the reviewers. Any product that may be evaluated in this article, or claim that may be made by its manufacturer, is not guaranteed or endorsed by the publisher.

## Abbreviations

BAL, Bronchoalveolar lavage; COVID-19, Coronavirus disease 2019; CRP, C-reactive protein; dsDNA, Double-stranded DNA; LDH, Lactate dehydrogenase; LPS, Lipopolysaccharide; MCH, Mean corpuscular hemoglobin; NET, Neutrophil extracellular trap; PBMC, Peripheral blood mononuclear cell; PCT, Procalcitonin; SARS-CoV-2, Severe acute respiratory syndrome coronavirus 2; TLR, Toll-like receptor.
