# Supplementary material for: Increased IL-26 associates with markers of hyperinflammation and tissue damage in patients with acute COVID-19
Source: Front Immunol. 2022 Nov 17;13:1016991. doi: 10.3389/fimmu.2022.1016991 (PMC9712219; doi:10.3389/fimmu.2022.1016991)
Supplement: Supplementary file 1 [file DataSheet_1.docx]

Supplementary Material

**Supplementary Table 1. Characteristics of the study subjects.**

|  | **COVID‑19 group (n = 49)** | **Control group (n = 27)** |
| --- | --- | --- |
| Age (median, range) | 61 years (25 – 90) | 43 years (25 – 61) |
| Sex (n, %)  Male  Female | 30 (61%)  19 (39%) | 9 (33%)  18 (67%) |
| Supplemental Oxygen (n, %)  Yes  No | 44 (90%)  5 (10%) | N/A |
| Hospitalization days  (median, range) | 8 (2 – 66) | N/A |
| Comorbidities (n, %)  Diabetes  Hypertension  COPD^a^  Asthma | 4 (8%)  11 (22%)  6 (12%)  5 (10%) | N/A |
| Smoking (n, %)  Yes  No | 2 (4%)  47 (96%) | N/A |
| Clinical laboratory parameters (mean ± SD)  CRP^b^ mg/L  Procalcitonin µg/L  LDH^c^ µkat/L  WBCs^d^ 10^9^/L | 53.04 ± 52.88  0.19 ± 0.25  5.13 ± 1.40  7.61 ± 2.40 | N/A |

N/A = Not available

^a^ Chronic obstructive pulmonary disease

^b^ C-reactive protein

^c^ Lactate dehydrogenase

^d^ White blood cells


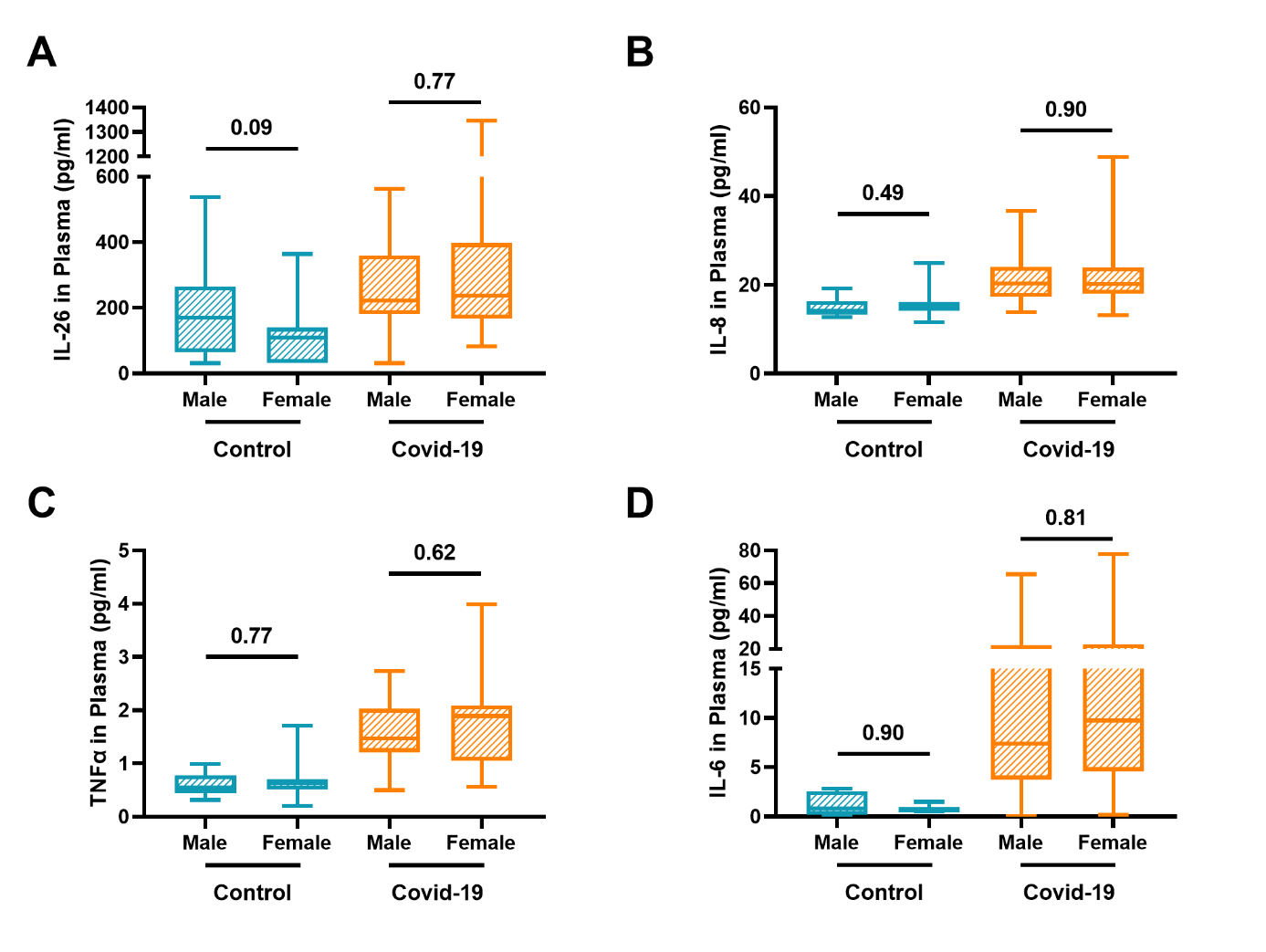


**Supplementary Figure 1. There are no gender differences in the plasma concentrations of IL‑26, IL‑8, TNFα, and IL‑6 of our patient material.** Gender comparisons of the plasma concentrations of **(A)** IL‑26, **(B)** IL‑8, **(C)** TNFα, and **(D)** IL‑6 in the COVID‑19 (orange) and Control (blue) groups tested by unpaired Mann-Whitney test.


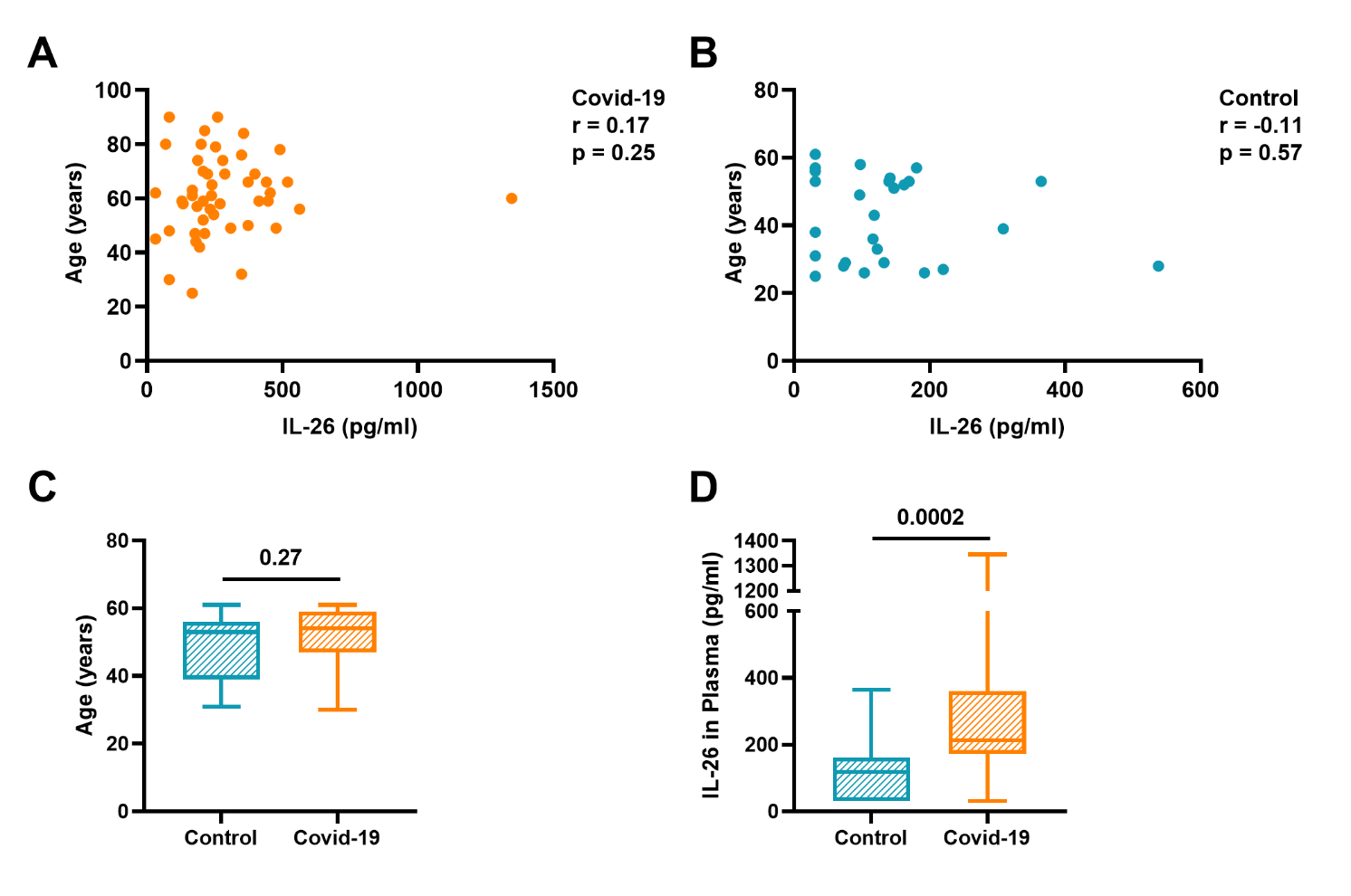


**Supplementary Figure 2. There are no age-dependent differences in the plasma concentration of IL‑26 of our patient material.** Spearman correlation analyses of the plasma concentration of IL‑26 with subject age in the **(A)** COVID‑19 (orange) and **(B)** Control (blue) groups, respectively. **(C)** Age comparison among a subgroup of age matched subjects (30-61 years of age) from the COVID‑19 (orange; n = 25) and Control (blue; n = 19) groups tested by unpaired Mann-Whitney test. **(D)** Comparison between the plasma concentration of IL‑26 among age matched subjects in the COVID‑19 and Control groups tested by unpaired Mann-Whitney test.

**
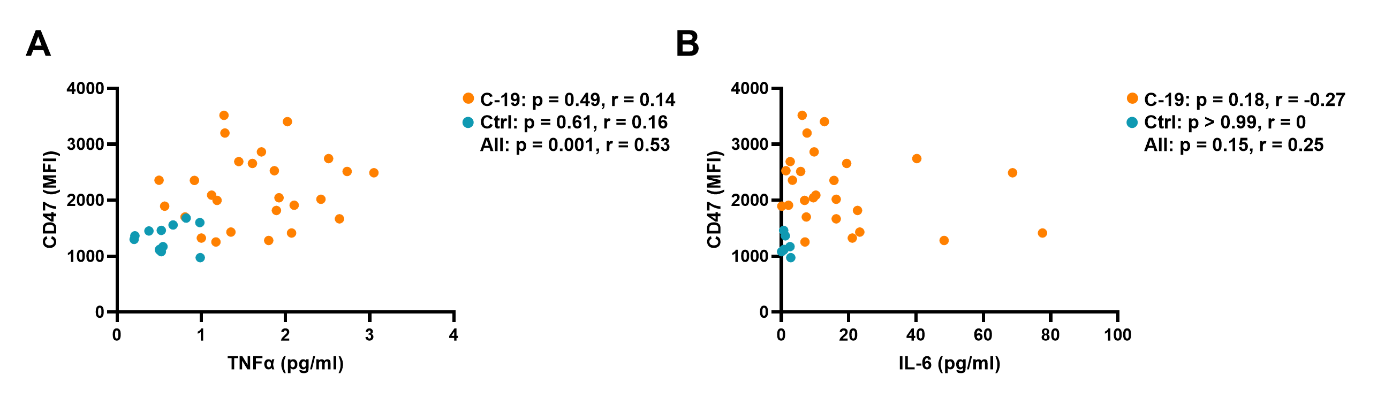
**

**Supplementary Figure 3. The plasma concentrations of IL‑6 and TNFα are not associated with the surface expression of CD47 on blood neutrophils in acute COVID‑19.** Spearman correlation analyses of the plasma concentrations of **(A)** TNFα and **(B)** IL‑6 with the surface expression of CD47 on blood neutrophils from the COVID‑19 (orange) and Control (blue) groups.

**Supplementary Figure 4. The plasma concentration of IL‑26 is not associated with the surface expression of CD11b or CD66b on blood neutrophils, nor with the percentage of CD49d^+^ blood neutrophils, in acute COVID‑19.** Spearman correlation analyses of the plasma concentration of IL‑26 with the surface expression of **(A)** CD11b and **(B)** CD66b on blood neutrophils, and with the percentage of **(C)** CD49d^+^ blood neutrophils, from the COVID‑19 (orange) and Control (blue) groups.

**Supplementary Figure 5. The plasma concentrations of IL‑6, IL‑8, and TNFα are not associated with the surface expression of CD11b or CD66b on blood neutrophils in acute COVID‑19.** Spearman correlation analyses of the plasma concentrations of **(A, D)** IL‑6, **(B, E)** IL‑8, **(C, G)** IL‑26, and **(C, F)** TNFα with the surface expression of **(A-C)** CD11b and **(D-F)** CD66b on blood neutrophils from the COVID‑19 (orange) and Control (blue) groups.

**Supplementary Figure 6. The plasma concentrations of IL‑6, IL‑8, and TNFα are not associated with the percentage of CD49d^+^ blood neutrophils in acute COVID‑19.** Spearman correlation analyses of the plasma concentrations of **(A)** IL‑6, **(B)** IL‑8, **(C)** TNFα with the percentage of CD49d^+^ blood neutrophils from the COVID‑19 (orange) and Control (blue) groups.

**Supplementary Figure 7. The plasma concentration of IL‑6 is only partially associated with markers of NET formation in acute COVID‑19.** Spearman correlation analyses of the plasma concentration of IL‑6 with the plasma concentration of **(A)** double-stranded DNA (dsDNA) and the plasma levels of **(B)** cell-free nucleosomes from the COVID‑19 (orange) and Control (blue) groups.


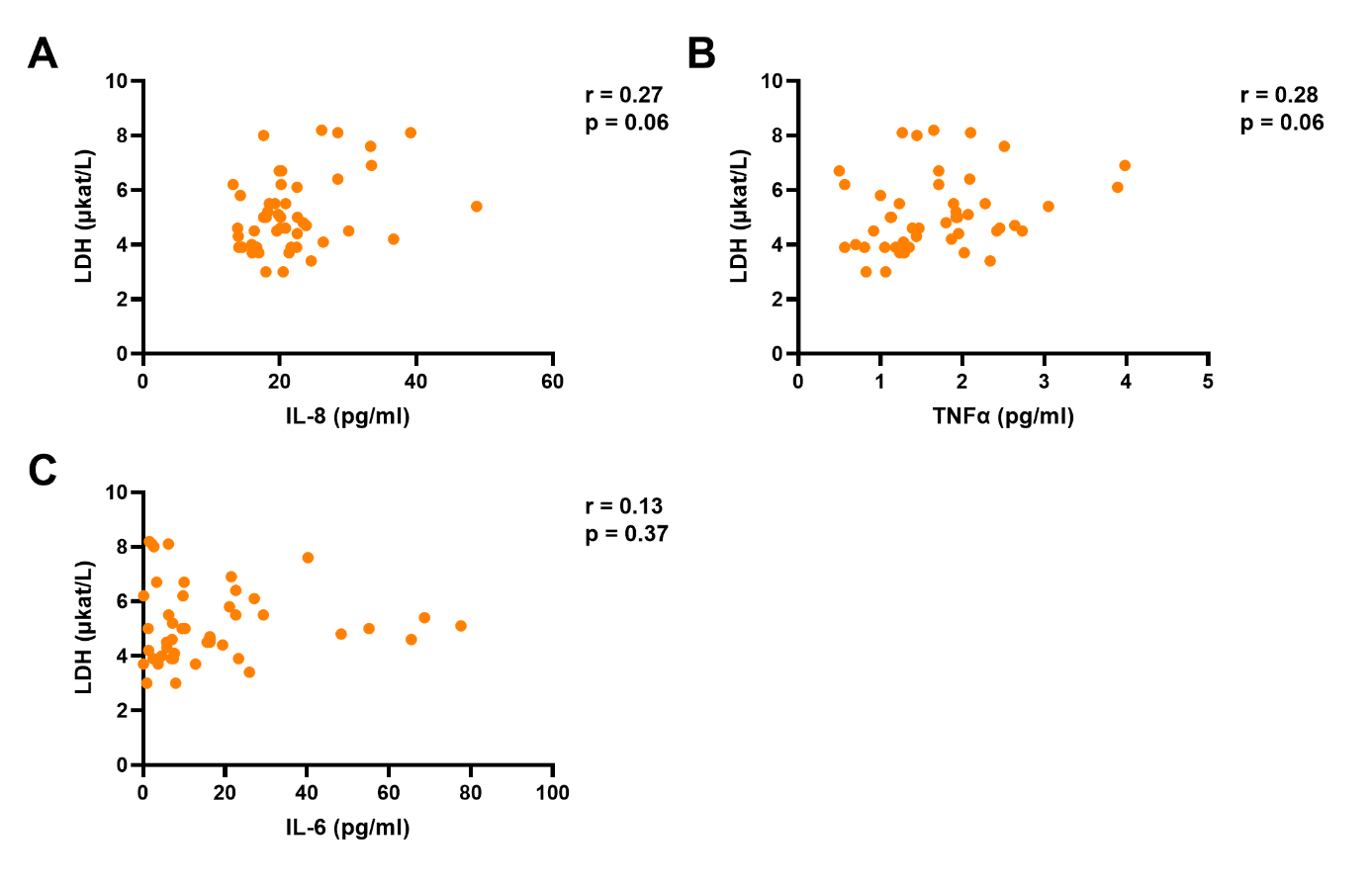


**Supplementary Figure 8. The plasma concentrations of IL‑8, TNFα, and IL‑6 are not associated with that of lactate dehydrogenase in acute COVID‑19.** Spearman correlation analyses of the plasma concentrations of **(A)** IL‑8, **(B)** TNFα, and **(C)** IL‑6 with that of lactate dehydrogenase (LDH) from the COVID‑19 group.


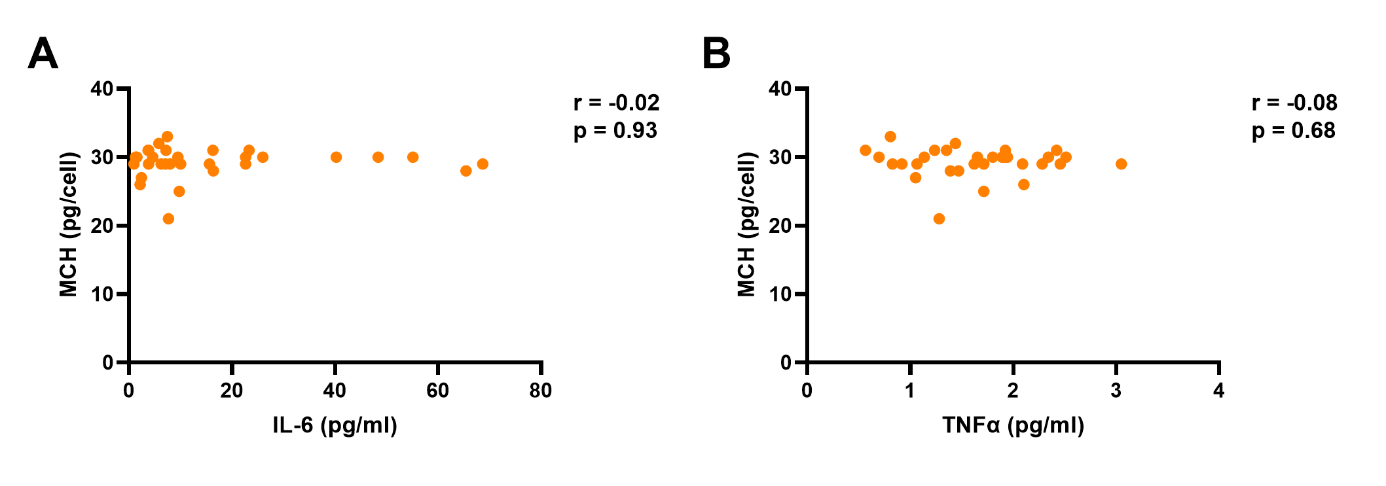


**Supplementary Figure 9. The plasma concentrations of IL‑6 and TNFα are not associated with the mean corpuscular hemoglobin in acute COVID‑19.** Spearman correlation analyses of the plasma concentrations of **(A)** IL‑6 and **(B)** TNFα with that of the mean corpuscular hemoglobin (MCH) from the COVID‑19 group.


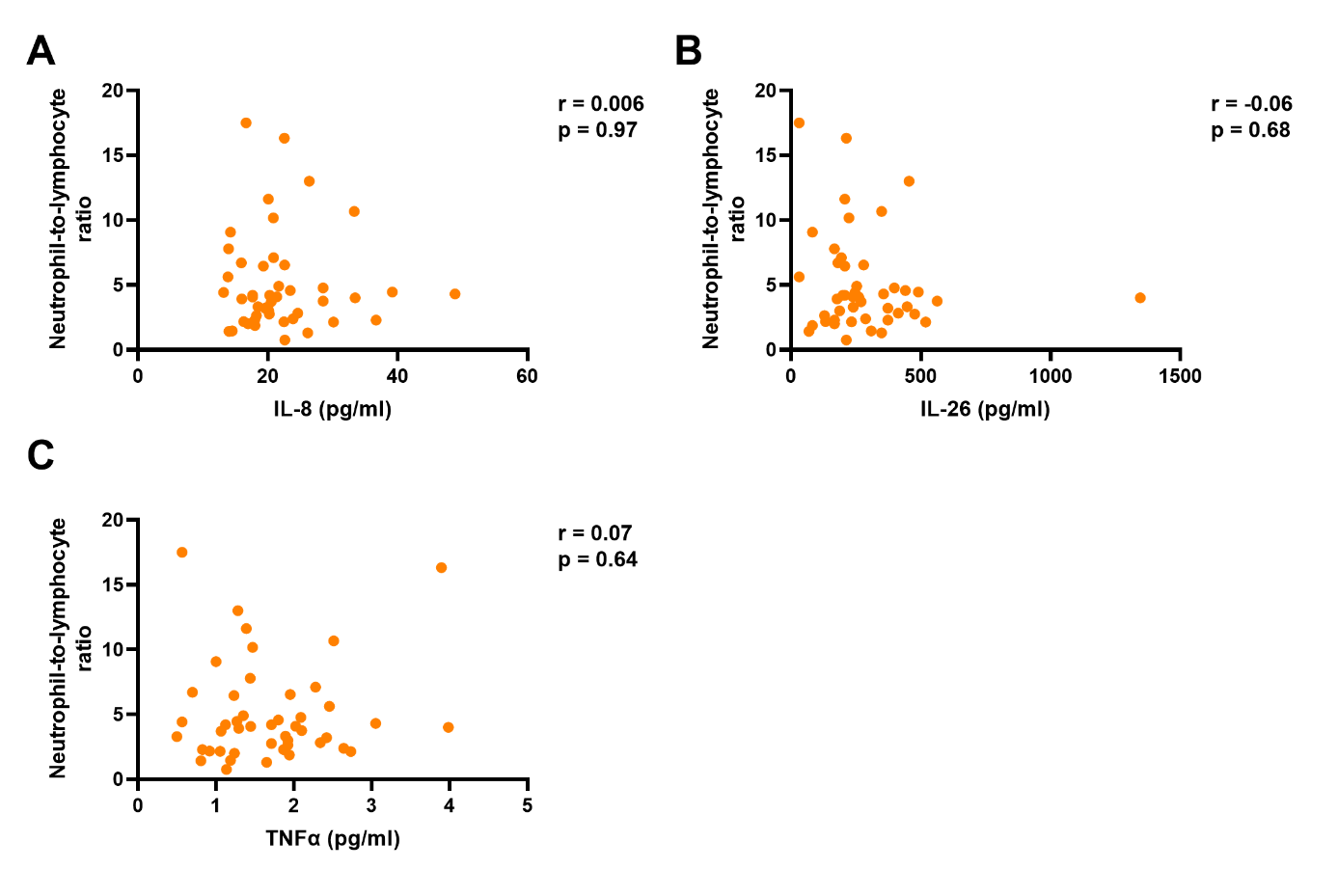


**Supplementary Figure 10. The plasma concentrations of IL‑8, IL‑26, and TNFα are not associated with the blood neutrophil-to-lymphocyte ratio in acute COVID‑19.** Spearman correlation analyses of the plasma concentrations of **(A)** IL‑8, **(B)** IL‑26, and **(C)** TNFα with the blood neutrophil-to-lymphocyte ratio from the COVID‑19 group.


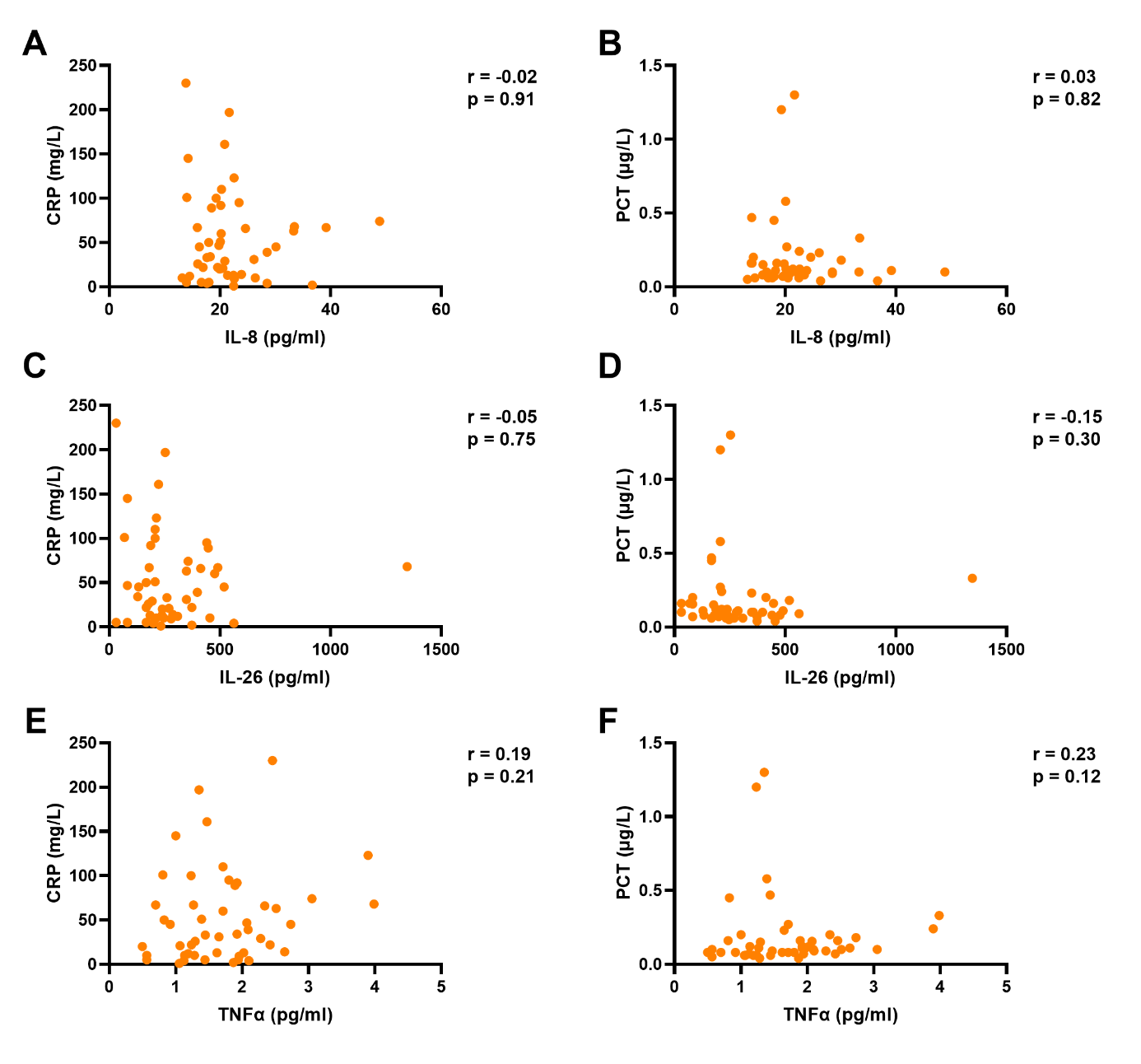


**Supplementary Figure 11. The plasma concentrations of IL‑8, IL‑26, and TNFα are not associated with those of C-reactive protein and procalcitonin in acute COVID‑19.** Spearman correlation analyses of the plasma concentrations of **(A-B)** IL‑8, **(C-D)** IL‑26, and **(E-F)** TNFα with those of **(A, C, E)** C-reactive protein (CRP) and **(B, D, F)** procalcitonin (PCT) from the COVID‑19 group.


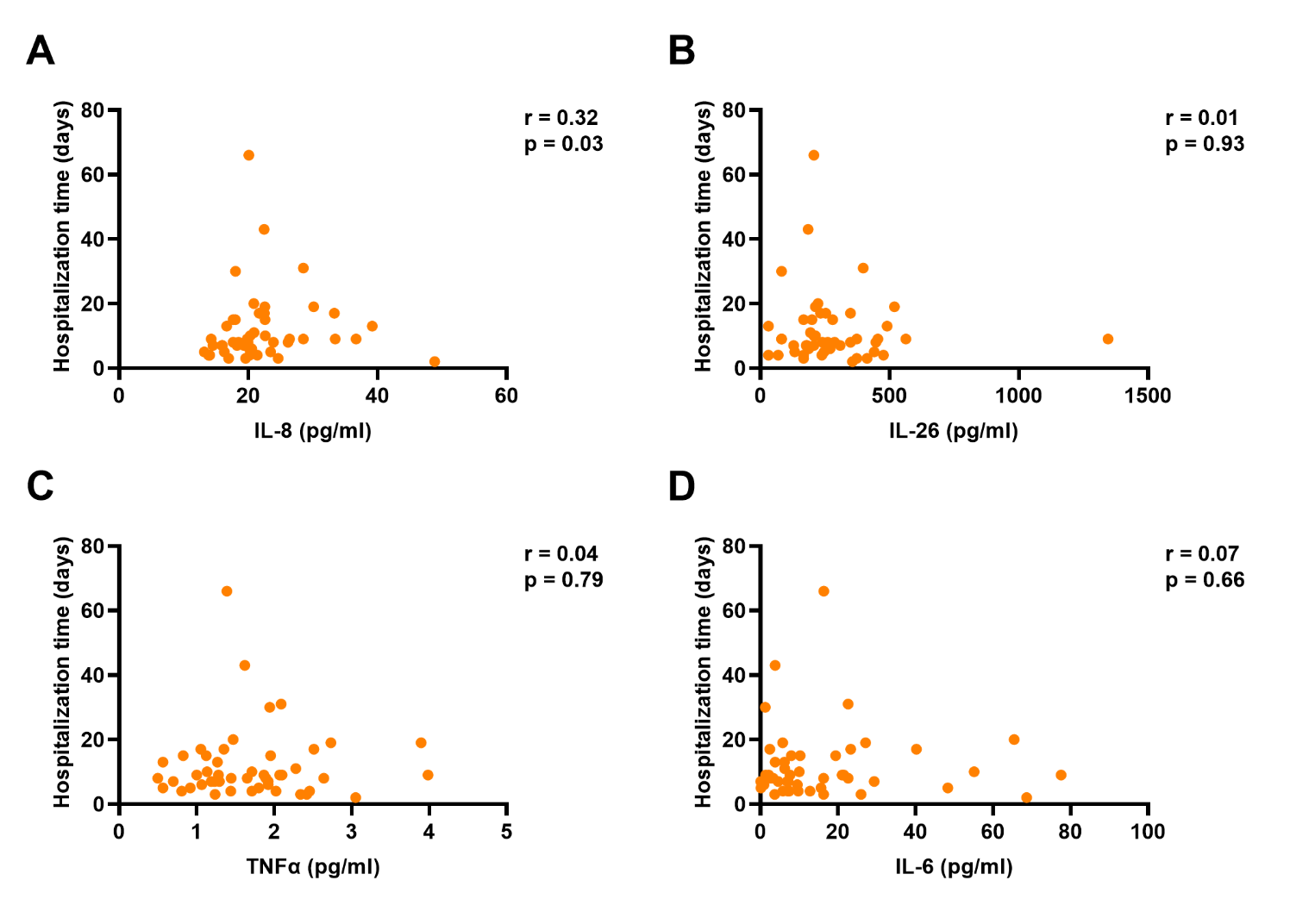


**Supplementary Figure 12. The plasma concentration of IL‑8 is associated with hospitalization time in acute COVID‑19.** Spearman correlation analyses of the plasma concentrations of **(A)** IL‑8, **(B)** IL‑26, **(C)** TNFα, and **(D)** IL‑6 with hospitalization time in the COVID‑19 group.
